# An overview of Zika virus genotypes and their infectivity

**DOI:** 10.1590/0037-8682-0263-2022

**Published:** 2022-09-30

**Authors:** Lucas Coêlho Bernardo-Menezes, Almerinda Agrelli, Ana Sofia Lima Estevão de Oliveira, Ronald Rodrigues de Moura, Sergio Crovella, Lucas André Cavalcanti Brandão

**Affiliations:** 1 Instituto Aggeu Magalhães, Fundação Oswaldo Cruz, Laboratório de Virologia e Terapia Experimental, Recife, PE, Brasil.; 2 Centro de Tecnologias Estratégicas do Nordeste, Laboratório de Materiais Nanoestruturados, Recife, PE, Brasil.; 3 Universidade Federal de Pernambuco, Departamento de Patologia, Recife, PE, Brasil.; 4 Institute for Maternal and Child Health IRCCS Burlo Garofolo, Department of Advanced Diagnostics, Trieste, Italy.; 5 University of Qatar, Department of Biological and Environmental Sciences, Doha, State of Qatar.

**Keywords:** Brazilian isolate, Congenital Zika syndrome, Mammalian cells, Sexual transmission route, Viral reservoir

## Abstract

Zika virus (ZIKV) is an enveloped, single-stranded RNA arbovirus belonging to the genus *Flavivirus*. It was first isolated from a sentinel monkey in Uganda in 1947. More recently, ZIKV has undergone rapid geographic expansion and has been responsible for outbreaks in Southeast Asia, the Pacific Islands, and America. In this review, we have highlighted the influence of viral genetic variants on ZIKV pathogenesis. Two major ZIKV genotypes (African and Asian) have been identified. The Asian genotype is subdivided into Southwest Asia, Pacific Island, and American strains, and is responsible for most outbreaks. Non-synonymous mutations in ZIKV proteins C, prM, E, NS1, NS2A, NS2B, NS3, and NS4B were found to have a higher prevalence and association with virulent strains of the Asian genotype. Consequently, the Asian genotype appears to have acquired higher cellular permissiveness, tissue persistence, and viral tropism in human neural cells. Therefore, mutations in specific coding regions of the Asian genotype may enhance ZIKV infectivity. Considering that mutations in the genomes of emerging viruses may lead to new virulent variants in humans, there is a potential for the re-emergence of new ZIKV cases in the future.

## INTRODUCTION

Zika virus (ZIKV) is a *Flavivirus* transmitted through the bite of female mosquitoes of the *Aedes*, *Culex*, and *Anopheles* genera^1^. Zika was first isolated from a Rhesus monkey in 1947 in Zika Forest, Uganda^2^. In 1954, the first case reported in humans was described on the African continent^3^. ZIKV was also detected in Asia in 1966 and has remained restricted to this region for almost five decades^4^.

In the early 2000s, ZIKV outbreaks were reported in regions of Southeast Asia, the Pacific Islands, and the Americas, with a proportional increase in infection rates. Outbreaks from Pacific Island and the Americas present higher numbers of cases^5^. In general, ZIKV had a higher epidemiological impact in tropical and subtropical countries once the mosquito *Aedes* spp. became a “cosmopolitan” vector, being widely distributed in tropical areas^1^. The first reported ZIKV outbreak occurred on Yap Island, Federated States of Micronesia, in 2007^6^. In 2013, ZIKV was associated with the development of Guillain-Barre syndrome (GBS) in the Pacific Islands of French Polynesia^7^. In 2016, Brazil recorded 440,000-1,300,000 suspected cases and 2,975 cases of ZIKV-associated microcephaly^8^, which led the World Health Organization to declare a worldwide state of public health emergency^9^. 

Acute ZIKV infections, known as Zika fever, generally result in mild illness in adults. The viral incubation period varies from 3 to 10 days, and most patients do not require hospitalization^5^. Zika fever is clinically characterized by fever, rash, fatigue, conjunctivitis, arthralgia, headache, myalgia, and retroorbital pain. These symptoms manifest in about 20-25% of symptomatic individuals. However, a small percentage of cases have been associated with neurological disorders in neonates (mainly microcephaly), a condition later named congenital Zika syndrome (CZS)^9^. 

Decades later, efforts of the scientific community to identify a vector control method, as well as vaccines and treatments to combat ZIKV infection, continue. Similarly, elucidating the pathophysiological mechanisms underlying this infection remain a challenge. During infection, host cells demonstrate morphological and molecular alterations^10^ ^,^ ^11^ that eventually culminate in mitotic abnormalities and cell death^12^, leading to tissue loss and neurological injury^13^. 

Many studies have shown that structural and nonstructural proteins are crucial components of viral pathogenesis^10^ ^,^ ^11^. However, it remains unclear which genetic factors of ZIKV may increase infection rate and virulence in humans. Here, we discuss the latest findings related to ZIKV genetic variants in terms of the infection process, cellular permissiveness, and tissue persistence.

### ZIKV genome and life cycle

The ZIKV genomic organization is similar among members of the *Flavivirus* genus (*Flaviviridae* family) such as dengue virus (DENV), yellow fever virus (YFV), and West Nile virus (WNV)^14^. The ZIKV genome consists of 10,794 nucleotides in a single-stranded positive-sense RNA that encodes a polyprotein of 3,424 amino acids and 10 proteins crucial for the viral life cycle^10^. ZIKV RNA has two untranslated regions (UTRs) and a single open reading frame (ORF). 

The 5′ and 3′ UTRs exhibit methylated nucleotides and non-polyadenylated forms, respectively, forming a loop structure. Moreover, the 5′ and 3′ UTRs have an essential function in virus replication. The 5′ UTR mediates the “start” signal for reading through the CAP AUG type 1 structure. Meanwhile, the 3′ UTR has a poly(A) tail that functions as a “stop” signal for the final step in polyprotein processing^15^ ^,^ ^16^. The ORF encodes three structural proteins (E, prM, and C) and seven nonstructural proteins (NS1, NS2A, NS2B, NS3, NS4A, NS4B, and NS5)^10^. 

ZIKV must undergo attachment, entry, replication, and exocytosis to successfully infect human cells. ZIKV cell attachment is mediated by attachment factors such as negatively charged glycosaminoglycans^17^. These molecules retain viral particles on the cell surface, providing conditions for membrane fusion. The entry process occurs via ZIKV envelope protein E^18^, which interacts with entry receptors in the host cell, such as C-type lectin^19^ and phosphatidylserine (PS) receptors^20^. These interactions cause conformational changes in the cell membrane and induce clathrin-mediated endocytosis, allowing the release of the viral genome into the cytoplasm^21^. 

Considering this, C-type lectin receptors, such as DC-SIGN (dendritic cell-specific intercellular adhesion molecule-3-grabbing non-integrin) and L-SIGN (liver/lymph node-specific intercellular adhesion molecule-3-grabbing integrin) recognize N-glycans linked to viral protein E, allowing viral entry^18^ ^,^ ^19^, whereas PS (present in the ZIKV envelope) is recognized by PS receptors, such as TAM (Tyro3, Axl, and Mer) and TIM (TIM1, TIM3, and TIM4)^20^.

ZIKV protein E is the largest antigenic glycoprotein in flaviviruses and plays a role in adhesion, recognition, and fusion to the host cell. The dimeric structure of protein E contains an ectodomain with three domains: DI, DII, and DIII^18^ ^,^ ^19^ ^,^ ^22^. DI has a structural function in that it acts as a binder and chemical support for other domains. DII interacts and promotes fusion on the cell membrane through a loop-shaped structure located on the support loop with DI^18^. DIII is an immunoglobulin-like domain with the capacity to bind extracellular receptors^23^ ^,^ ^24^. Protein E contains a glycosylation site in an asparagine residue (Asn154), which may be associated with ZIKV virulence. 

This pattern of N-glycosylation is conserved among DENV, YFV, and WNV. In DENV, glycosylation follows the Asn154 and Asn67 residues^19^. According to Wen^18^, N-glycosylated residues on protein E may enhance ZIKV infectivity by increasing the affinity of protein E to the entry receptors. 

Once inside the cell, the low pH within the endosome enables the native state of protein E, which subsequently fuses to the endosome membrane and releases the viral RNA into the cytoplasm. Once in the cytoplasm, ZIKV undergoes particle assembly, followed by RNA replication and translation into viral proteins^25^. During maturation, newly assembled viral particles enter the endoplasmic reticulum (ER) and acquire PS. Viral particles then migrate from the ER to the Golgi complex where viral maturation occurs^26^. This process is mediated by the protein furin in the host, which cleaves the prM protein into the “pr” and “M” portions^22^ ^,^ ^25^. Finally, new mature ZIKV viral particles are released into the extracellular environment^22^.

### ZIKV genotypes

To date, two major ZIKV genotypes have been identified: African and Asian. The African-ZIKV genotype has caused sporadic or recurrent infections in West African countries, with clinical manifestations of fever, conjunctivitis, and myalgia^3^ ^,^ ^27^. Nevertheless, the Asian-ZIKV genotype has circulated in Southeast Asia, the Pacific Islands, and the Americas, causing major outbreaks characterized by fever, arthralgia, conjunctivitis, CSZ, GBS, and ophthalmological anomalies^6^ ^,^ ^7^ ^,^ ^9^ ^,^ ^28^ ^.^ Through the timespan of these major outbreaks, it has been reported that the number of people with severe symptoms has increased as the Asian-ZIKV epidemic has disseminated among continents^29^ ^,^ ^30^. 

**FIGURE 1:**
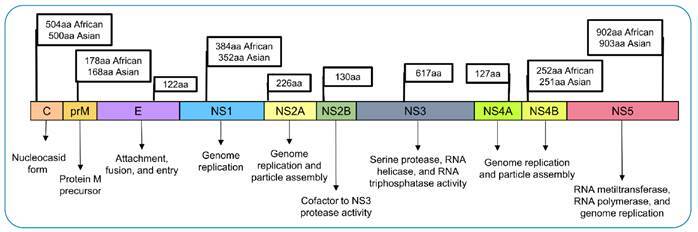
ZIKV polyprotein from the African- and Asian-ZIKV genotypes, structural proteins (C, prM, and E) and nonstructural proteins (NS1, NS2A, NS2B, NS3, NS4A, NS4B, and NS5) as well as their sizes, in amino acids (aa), and functions in the viral cycle.

The African-ZIKV genotype is subdivided into East African and West African strains. The Asian-ZIKV genotype is subdivided into Southwest Asia, Pacific Island, and American strains^31^. The African and Asian genotypes exhibit few different amino acid sequences^14^. Nevertheless, they share subcellular locations in host cells and protein function. ZIKV polyprotein from African and Asian genotypes are schematized in Figure 1. 

Shrivastava^32^ and Collins^33^ observed phylogenetic diversity in both African and Asian-ZIKV genotypes as well as insertions/deletions in their viral genomes. Moreover, Barzilai and Schrago^34^ posited that ZIKV spread may be associated with nonsynonymous mutations as a consequence of the viral evolution rate. Overall, Asian genotypes (lineages from Malaysia, Cambodia, and America) show higher genetic variants and single nucleotide variants in the viral genome than African genotypes (East and West African lineages)^32^ ^-^ ^34^. 

According to Collins^33^, the African genotype exhibits fewer synonymous mutations (G3589T, G3589A, C5080A, and C5080T) and nonsynonymous mutations (G3299A, A3300G, and T5079A) than the Asian genotype, with 18 nonsynonymous mutations and only one synonymous mutation. This may explain why both African strains remained restricted to the African continent^32^.

Taking into consideration the coding region sequences in the Asian genotype, Faria^35^ and Ye^14^ found great genetic similarities between ZIKV strains from the Pacific Islands and the Americas. However, these ZIKV strains exhibited a phylogenetic distance of decades compared to strains from Malaysia, which were later identified as a Southeast Asian strain^35^. In addition to phylogenetic differences, dissimilar nucleotides were also found between strains from the Pacific Islands and Malaysia^14^ ^,^ ^35^, indicating that these Asian strains do not share the same lineage. ZIKV strains from the Pacific Islands and Americas constitute only one lineage within the Asian genotype^14^ ^,^ ^35^. Among the lineages of the Asian genotype, Malaysian strains sampled in 1966 were the oldest^35^.

Ye^14^ suggested that the American strain constitutes a new clade within the Asian-ZIKV genotype. Reports also indicated a common origin among ZIKV strains from Micronesia, French Polynesia, and Brazil during outbreaks in 2007, 2013, and 2016, respectively^14^ ^,^ ^31^ ^,^ ^36^. However, many reports indicate that there are variations among amino acids throughout the Asian-ZIKV genome, which can lead to viral adaptations (Table 1). In this context, a study conducted by Kawai^31^ evaluated the pathogenicity of Southern Asian, Pacific Island, and American strains *in vitro* and *in vivo*. It has been shown that the American strain induces strong pathogenicity^31^.

**TABLE 1:** Characterization of nonsynonymous mutations on the Asian-ZIKV genome.

Protein	Polyprotein position	Isolates	Substitution*	Reference
C	81	Malaysia	I → M	^32^
	81	Thailand	I → M	^32^
	81	México	I → M	^32^
	81	Honduras	I → M	^32^
prM	139	French Polynesia	S → N	^40^
	139	Brazil	S → N	^40^
	168	Malaysia	D → K	^32^
E	356	China	D → N	^43^
	451	Colombia	D → E	^32^
	451	Panama	D → E	^32^
	456	Brazil	R → K	^37^
	763	China	V → M	^42^
	600	Thailand	A → E	^24^
	620	Puerto Rico	V → L	^32^
	620	Malaysia	L → V	^32^
	683	Thailand	E → K	^24^
	691	Malaysia	Y → H, H → Y	^32^
NS1	852	Panama	F → S	^33^
	969	Honduras	Y → S	^33^
	1033	Colombia	S → N	^32^
	1033	Porto Rico	S → N	^32^
	1033	Panama	S → N	^32^
	1143	Brazil	V → M	^37^
NS2A	1176	Brazil	T → I, I → T	^37^
	1180	Brazil	I → Y, T → I	^37^
	1263	Brazil	V → A	^37^
	1263	Malaysia	V → A	^32^
	1263	Thailand	A → V	^32^
	1303	Panama	A → V	^32^
	1303	Malaysia	V → A	^32^
	1327	Brazil	M → V, V → M	^37^
	1370	Honduras	G → R	^33^
NS2B	1411	Cambodia	I → T	^38^
NS3	1594	Brazil	H → Y, Y → H	^37^
	1857	Thailand	H → Y	^24^
NS4B	2295	Brazil	I → T	^37^
	2857	China	E → D	^41^

*I: isoleucine; **M:** methionine; **S:** serine; **N:** asparagine; **D:** aspartic acid; **K:** lysine; **E:** glutamic acid; **R:** arginine; **V:** valine; **L:** leucine; **Y:** tyrosine; **T:** threonine; **A:** alanine; **G:** glycine; **H:** histidine.

In addition, Strottmann^37^ and Regla-Nava^38^ suggested that mutations in NS2A (A117V) and NS2B (I39V) from Asian strains may impact the infectivity of mammalian and insect cells. Using an *in silico* approach, mutations with relevant structural impacts were found in protein C (I80T) and NS2A (K113F, A143V, and I199V) of circulating ZIKV strains from French Polynesia, Brazil, and Colombia^39^. Strottman^37^ detected nonsynonymous mutations in proteins E (R166K), NS1 (V349M), NS2A (I30T, T34I, V117R, and V1181M), NS3 (H92Y), and NS4B (I26T) of three ZIKV isolates from Brazilian regions.

Other *in vitro* and *in vivo* studies have been conducted to elucidate the impact of nonsynonymous mutations on the Asian-ZIKV genome. Yan^40^ demonstrated that the mutation S139N in prM of the Asian genotype may contribute to the development of CZS. This mutation in the prM protein was detected before the outbreak in French Polynesia, and it remained stable during ZIKV spread until the outbreak in the Americas in 2015^40^. In the viral protein NS4B, the substitution E2587D was observed in an Asian strain from China, in 2016^41^. Moreover, two substitutions in protein E (D67N and V473M) may have increased ZIKV replication and neurovirulence as well as its transmission during pregnancy and viremia after the American epidemic^42^ ^,^ ^43^. In an Asian isolate from a Thai patient in 2021, unique nonsynonymous mutations were detected in proteins E (A310E and E393K) and NS3 (H355Y)^24^. These findings suggest that after the outbreak in French Polynesia and before the outbreak in the Americas, ZIKV strains might have mutated and acquired higher infectivity. 

Moreover, Li^44^ proposed that proteins E, C, and prM contribute to Asian-ZIKV attachment, permissiveness, and cytopathic effects in human glial cells. In addition, NS2A recruits unprocessed proteins to be cleaved by NS2B/NS3 serine-protease at the E-prM-C site^45^. NS2A and NS4B also play a role in the assembly of new particles^11^. Haddow^36^ demonstrated that ZIKV genotypes can exhibit different N-glycosylation sites, whereas Bos^46^ found new glycosylated residues in protein E (I152, T156, and H158) in Brazilian ZIKV strains. Highly glycosylated residues may influence ZIKV attachment, entry, and fusion with host cells^46^. 

### Cellular permissiveness of ZIKV

**FIGURE 2:**
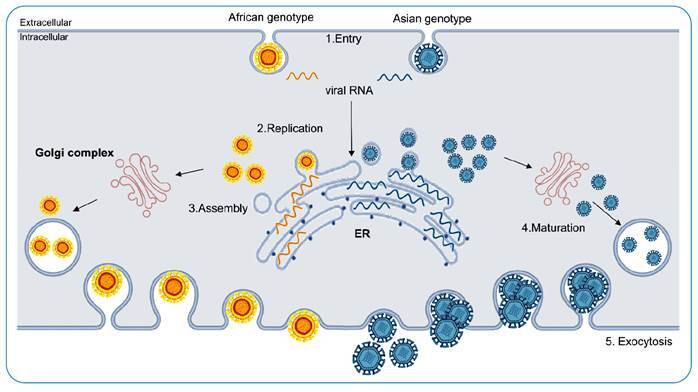
Permissiveness and replication cycle of the ZIKV genotypes in host cell.

 ZIKV is known to infect different hosts, ranging from mosquitoes to mammals, as well as many cell types and tissues (Figure 2). Rat mesenchymal stem cells, mouse embryonic fibroblasts, murine macrophages, monkey kidneys, and mosquito larvae cells are some non-human cellular models that have been described as susceptible to ZIKV entry, replication, and release^47^. 

The entry processes of African and Asian genotypes in humans share a highly conserved mechanism that requires clathrin-mediated endocytosis^21^. Among human cells, ZIKV is known to infect dermal fibroblasts^48^, fetal neurons^49^, primary Hofbauer^50^ and mesenchymal stem cells^47^, epidermal keratinocytes^48^, fetal cortical astrocytes^49^, primary trophoblasts^50^, embryonic kidney cells^47^, and sperm cells^51^. Furthermore, some types of innate immune cells (such as primary monocytes and plasmacytoid dendritic cells) have been identified as permissive to viral infectivity^30^. 

During ZIKV infection, the skin cells mediate an early innate immune response^48^. *In vitro* studies have evaluated the persistence of ZIKV infection in human skin cells in an attempt to understand the infection route following mosquito bites in mammalian hosts. Hamel^52^ observed that human epidermal keratinocytes, dermal fibroblasts, and immature dendritic cells were fully permissive to French Polynesia isolates. However, Hou^26^ showed that fibroblasts and epidermal human lineages did not display any differences in permissiveness, infection rate, and replication modes between isolates from Uganda and Puerto Rico. 

According to Hou^26^, immunological cells did not demonstrate a difference in permissiveness between African- and Asian-ZIKV genotypes. However, Osterlund^53^ observed differences in replication rates among these genotypes, although both showed great replication in human dendritic cells. Unlike the African genotype, viral replication in the Asian genotype is attenuated in human macrophages^53^. These findings suggest that the Asian-ZIKV genotype may use immunological cells as a viral reservoir.

### Tissue persistence and viral tropism

During ZIKV infection, some cells and tissues may become viral reservoirs, contributing to the dissemination of Asian-ZIKV to nearby tissues. It was observed *in vitro* that both ZIKV genotypes have the capacity to infect human peripheral blood mononuclear cells^26^, indicating that these cells may act as an “entry door” for ZIKV spread. 

Moreover, ZIKV-infected monocytes exhibited a quicker transmigration process than cell-free viruses on endothelial barriers in studies using *in vitro*, *in vivo*, and *ex vivo* models^30^. ZIKV-infected mast cells were also detected *in situ* in the placental tissue of pregnant Brazilian women^54^. These reports indicate that ZIKV-infected immunological cells might circulate throughout the host’s blood tissue, promoting Asian-ZIKV spread and contributing to vertical transmission. 

Asian-ZIKV has also been found to be transmitted by the sexual route. For instance, Rashid^55^ observed the infection and replication of ZIKV (isolates from Puerto Rico) in primary human Sertoli cells *in vitro*, confirming ZIKV persistence in the reproductive tract and high cellular permissiveness. In addition, Matulasi^51^ demonstrated that ZIKV isolates from French Polynesia infect reproductive and somatic testicular cells *in vitro*, as well as, replicates in human testes *ex vivo*. These studies suggest that American ZIKV strains can replicate in the male reproductive system. 

In this context, ZIKV-infected sperm cells can also infect tissues of the female reproductive system during sexual encounters. Using an *in vitro* approach, studies have demonstrated that human primary endometrial^56^, Hofbauer, and trophoblast cells^50^ are vulnerable target cells of American ZIKV strains. Thus, once ZIKV infects and replicates in reproductive tissues, it poses a risk at different stages of pregnancy.

Considering that neuronal progenitor cells and glial cells, which are crucial for neurogenesis, can also be targeted by ZIKV, the central nervous system (CNS) inflammatory process during gestation can significantly impact brain development. Hence, diverse studies have shown positive tropism between ZIKV genotypes and cells in the CNS. Li^57^ demonstrated that both African and Asian genotypes can infect and replicate in neurons and glial cells *in vitro*. In parallel, *in vitro* astrocytes have a good tolerance for high viral load rates for both viral genotypes^49^. However, according to Goodfellow^58^ and Aguiar^59^, loss of cellular proliferation, neuronal migration, and abnormal extracellular matrix have been observed only in infections caused by the Asian genotype. In addition, Cugola^60^ proposed that ZIKV strains that circulate in Brazil can trigger autophagy and apoptotic pathways, leading to cell death in cortical progenitor cells.

Thus, compared to African isolates, Brazilian ZIKV isolates exhibited higher neurotropism for neural cell lineages. These data led us to believe that the Asian genotype has greater virulence because its strains have accumulated large nonsynonymous mutations over the time of dissemination.

## CONCLUSIONS

We gathered information on the genetic variants of ZIKV and their influence on the viral life cycle, cellular permissiveness, and tissue persistence. Based on the reviewed papers, we found that nonsynonymous mutations in the ZIKV genome may increase viral entry, RNA replication, particle assembly, and viral load. Considering that mutations in the genomes of emerging viruses may lead to new virulent variants in humans, this might be a possibility for the future re-emergence of new cases. Further *in vitro* and *in vivo* experiments are required to better evaluate these mutations.
